# The Ins and Outs of Rust Haustoria

**DOI:** 10.1371/journal.ppat.1004329

**Published:** 2014-09-11

**Authors:** Diana P. Garnica, Adnane Nemri, Narayana M. Upadhyaya, John P. Rathjen, Peter N. Dodds

**Affiliations:** 1 Research School of Biology, Australian National University, Canberra, Australian Capital Territory, Australia; 2 Division of Plant Industry, Commonwealth Scientific and Industrial Research Organisation (CSIRO), Canberra, Australian Capital Territory, Australia; Duke University Medical Center, United States of America

Rust diseases caused by fungi of the order Pucciniales afflict a wide range of plants, including cereals, legumes, ornamentals, and fruit trees, and pose a serious threat to cropping systems and global food security. The obligate parasitic lifestyle of these fungi and their complex life cycles, often involving alternate hosts for the sexual and asexual stages, also make this group of pathogens of great biological interest. One of the most remarkable adaptations of rust fungi is the specialized infection structure that underpins the sustained biotrophic association with hosts; the haustorium (Figure 1A and C). This organ forms after penetration of the wall of a live host cell, expanding on the inner side of the cell wall while invaginating the surrounding host plasma membrane (Figure 1C). Through haustoria, the pathogen derives nutrients from the host and secretes virulence proteins called effectors, which are believed to be the key players that manipulate the physiological and immune responses of host cells [1]–[4]. Analogous terminal feeding structures have independently evolved in other organisms such as the haustorium in powdery mildews (ascomycetes) and downy mildews (oomycetes, not true fungi), and the arbuscules in arbuscular mycorrhizae, suggesting that such architecture represents a successful adaptation of these organisms to interact with their respective host plants [5], [6].

**Figure 1:**
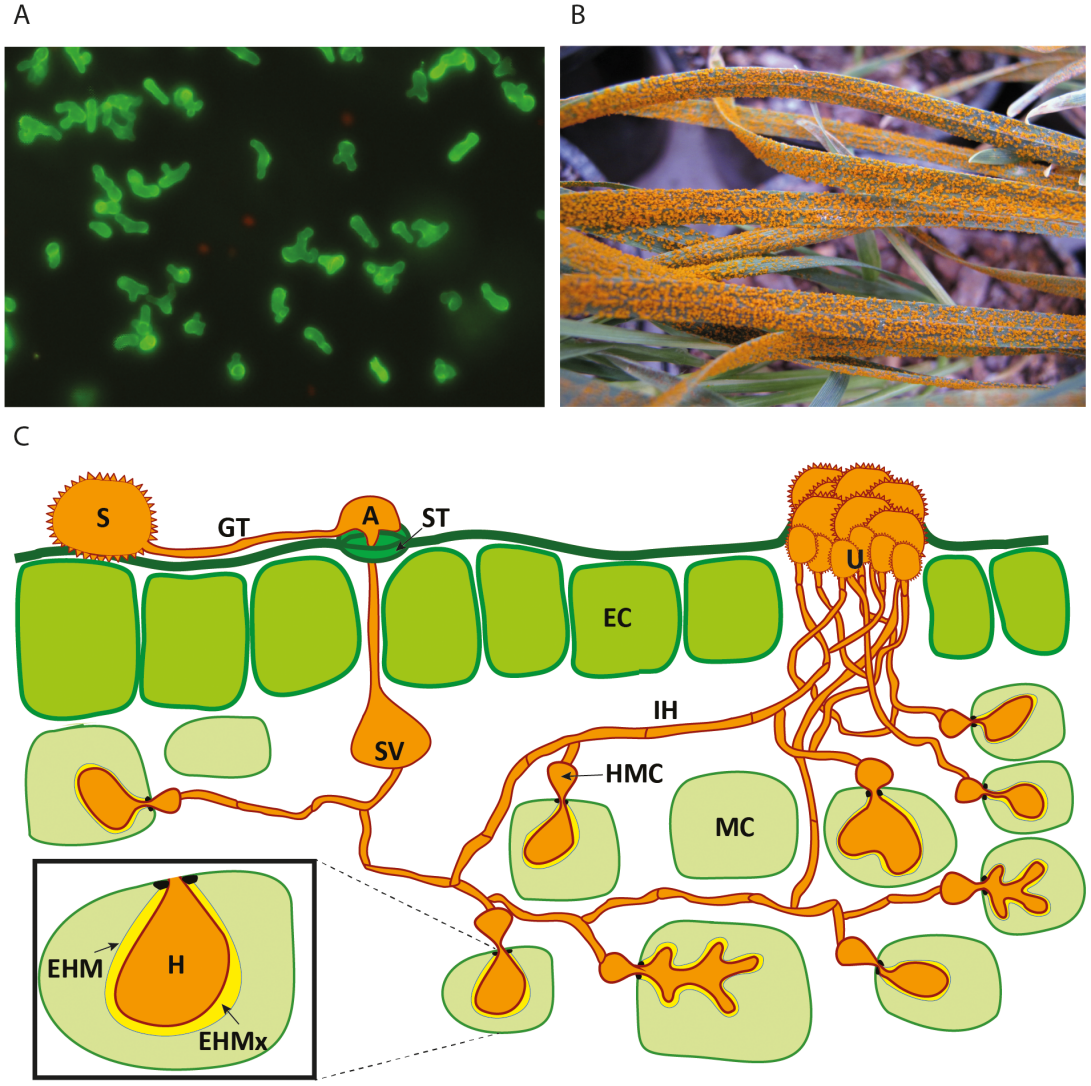
Schematic representation of the developmental phases of rust infection on wheat and example of macroscopic symptoms. A) Confocal microscopy of isolated haustoria from *Pst*-wheat infected tissue [34] stained with the lectin Concanavalin A (which has affinity for α-D-mannosyl and α-D-glucosyl groups present in various sugars, glycoproteins, and glycolipids), conjugated to Alexa Fluor 488. B) Massive uredospore production at advanced stages of the stripe rust disease on wheat seedlings. C) Representation of the asexual cycle of *Puccinia* spp. on wheat. The dikaryotic uredospore (S) lands on the leaf surface and produces a germination tube (GT) within 6 hours. Subsequently, it produces an appresorium (A) over the stomatal aperture and enters to the leaf interior through the stoma (ST), where it differentiates into a substomatal vesicle (SV). Primary infection hyphae (IH) propagate through the leaf, and once in contact with mesophyll cells, haustorial mother cells (HMC) differentiate. These penetrate the host mesophyll cell (MC) wall to form the haustorium (H). The haustorium remains separated from the host cell cytoplasm by the extrahaustorial matrix (EHMx) and the host-derived extrahaustorial membrane (EHM). After the establishment of the first haustorium, secondary hyphae develop, colonize the intercellular spaces, and give rise to more HMCs and haustoria. The cycle is completed within 10–11 days, when the invasive hyphae form sporogenous basal cells in the uredia (U) and thousands of new infective uredospores erupt through the leaf epidermis.

## Rust Haustoria Possess a Specialised Metabolism

The primary disease-causing stage of the rust life cycle is the asexual stage. Dikaryotic uredospores germinate on the leaf surface and then colonize the leaf tissue to establish the biotrophic interaction, which can be very long-lasting (Figure 1C). Ultimately, the infection gives rise to sporulating pustules that release vast numbers of new spores that can repeat the infection cycle through the growing season (Figure 1B, C). Early ultrastructural studies of dikaryotic rust infection processes showed that haustorium formation begins when a haustorial mother cell (HMC) (Figure 1C) differentiates from intercellular hyphae by laying down a septum near the hyphal tip [7]. During haustorium formation, the cytoplasmic contents of the HMC, including the two haploid nuclei, migrate into the haustorium through the neck structure, leaving the HMC enucleate and highly vacuolated. The HMC septum undergoes complex changes during host wall penetration and haustorial maturation, including occlusion of the central pore, thereby preventing continuity of the cytoplasmic contents throughout the hyphae [7]. Thus, the HMC and haustorium are separated from the hyphae, which may aid the development of independent transcriptional and metabolic programs in these cells.

The ability to purify rust fungi haustoria from infected plant tissue (Figure 1A) enabled the first analysis of haustorial gene expression, conducted in the bean rust fungus *Uromyces fabae* (*Uf*) [8]. This work identified several genes apparently involved in nutrient acquisition, including genes encoding a hexose transporter, HXT1 [2], and three amino acid transporters, AAT1, AAT2, and AAT3 [1], [9], [10]. Immunolocalization studies showed the exclusive localization of HXT1 and AAT2 at the haustorial plasma membrane [1], [2], while biochemical studies revealed that AAT1 and AAT3 function as proton-dependent transporters with preference for histidine/lysine and leucine/methionine/cysteine respectively [9], [10]. These studies provided the first compelling evidence for involvement of haustoria in nutrient uptake.

Since then, the emergence of high-throughput DNA and mRNA sequencing has greatly increased our understanding of the metabolic function of the haustorium. For instance, the transcriptomic analysis of isolated haustoria from wheat stripe rust *Puccinia striiformis* f. sp *tritici* (*Pst*), indicated that they are active in uptake of sugar, amino acids, nitrogen, and other nutrients through high expression of transmembrane transporters, and also in incorporation of these nutrients into biosynthetic and energy-manufacturing pathways for their utilization in fungal development [11]. This is in contrast to germinating stripe rust spores, which show expression patterns consistent with acquisition of energy from stored compounds. The haustorial transcriptomes from other rusts, such as the common bean and soybean rust pathogens *Uromyces appendiculatus* and *Phakopsora pachyrhizi* [12], and the wheat stem rust *Puccinia graminis* f. sp *tritici* (*Pgt*) [13], [14], show similar expression patterns, suggesting that rust haustoria share similar mechanisms to exploit host-derived resources. The four sequenced rust genomes, including the *Puccinia* species, *Pgt* and *Pst* [13], [15], [16], and the two *Melampsora* species, *Melampsora larici-populina* and *Melampsora lini* [13], [17], lack genes encoding key assimilatory enzymes for inorganic nitrate and sulphur, suggesting that rust pathogens obtain the reduced versions of these nutrients from plant cells.

## Haustoria Produce and Deliver Effectors to the Host Cytoplasm

Seminal studies on the bean and flax rust pathogens provide support for the idea that haustoria of rust fungi are responsible for the production and secretion of effectors, with a number of these proteins targeted to the host cytoplasm where they are thought to promote the infection. Rust transferred protein 1 (RTP1) from *Uf* and its homologue from *Uromyces striatus* were the first such proteins proven to be expressed specifically in the haustorium and transferred to the host cytoplasm during a compatible biotrophic interaction [8], [18]. RTP1 shares similarity with cysteine protease inhibitors and can inhibit proteolytic activity in yeast culture supernatants, so may act to inhibit host defence-associated proteases [19]. It can also form aggregates and filamentous-like structures inside the extrahaustorial matrix and the host cytoplasm, which may have a structural role in stabilizing the host cell allowing accommodation of the haustorium [20]. Emerging transcriptomic and genomic data from a range of rust fungi have identified RTP1 homologues in at least 13 rust species, suggesting that this protein could play an important role in the biotrophic lifestyle [19].

Four avirulence (*Avr*) genes, which encode effectors that are recognised by immune receptors encoded by host resistance (*R*) genes, have been identified in the flax rust *M. lini* [3], [21], [22]. All encode small secreted proteins that are expressed in haustoria and are recognised in the host cytoplasm, implying that these proteins are delivered into the host cell during infection. This was confirmed by direct visualisation of the effector AvrM inside infected flax cells [23]. This study also found evidence that at least some effectors can be taken up into the host cytosol independently of a specialized pathogen delivery system, since secreted AvrM and AvrL567 expressed by tobacco cells accumulated in the cytosol despite being targeted efficiently to the plant secretory system. Structural and functional studies of AvrM revealed a dimeric protein with intrinsic membrane-binding properties, which possesses a conserved hydrophobic surface patch required for pathogen-independent internalization [23], [24]. Although AvrM can bind negatively-charged phospholipids, this is not essential for its transport across the plant plasma membrane [24]. Overall, the mechanisms of effector delivery from rusts and other filamentous pathogens remain unknown and are the subject of much debate [5], [6]

## Rust Haustoria Express Many Effector Candidates

The characterisation of RTP1 and Avr proteins implied the existence of a class of rust effectors delivered into host cells from haustoria, some of which could become targets of recognition by immune receptors. Over 30 *Avr* specificities have been described in flax rust, and around 50 in each of *Pgt*, *Pst*, and *Puccinia triticina*, suggesting large families of such effectors. Indeed, genomic and transcriptomic studies on rust fungi have revealed large sets (500 to 1,500) of potential effector genes. In contrast to effectors in some other filamentous plant pathogens, such as the RxLR and crinkler class effectors of oomycetes [5], no conserved amino acid motifs are widely present in these proteins [16], [25]. In the absence of defined and conserved hallmarks in the sequences of effector genes, their prediction has been based on three main criteria: (1) presence of a secretion signal, (2) lack of transmembrane domains, and (3) expression in haustoria or infected tissue. For example, in the genomes of *Pgt*, *Pst*, *M. larici-populina*, and *M. lini* [13], [15], [17], about 8% of their predicted proteomes corresponds to candidate effectors that fulfil these criteria. Infection tissue-specific transcriptomes of these pathogens [11], [13], [26] and other rusts, including *Uf* [27], have identified large numbers of predicted effectors expressed in planta. More recently, haustoria-specific transcriptomic data detected expression of 70% of the predicted in planta effector complement in the haustorium of *Pst* (Jackson, et al. unpublished) and 58% in *Pgt* [14], lending additional support to the idea that the haustorium is the main source of effector proteins. Sperschneider, et al. (2014) [28] used an alternative, unbiased approach for effector prediction based on the comparison of 174 fungal genomes and the classification of genes into families associated with pathogenicity. This study revealed a cluster of proteins enriched in secretion signals, small amino acids and cysteine residues, confirming that these are useful criteria for effector prediction. The generation of lists of candidate effectors is an important first step that precedes functional assays to uncover their contributions to pathogenicity.

## Evolutionarily Diverged Effector Candidates May Control Host Specificity

Avirulence genes often exhibit high levels of polymorphism and display signatures of diversifying selection [3], [22], [29] as a result of antagonistic co-evolution with plant defences. For instance, positively selected polymorphic residues in AvrL567 are exposed on the protein surface and are responsible for differences in recognition specificity by host immune receptors [30], explaining the underlying molecular basis driving diversifying selection of this gene family to escape recognition. Likewise, AvrM is recognised by direct interaction with the corresponding M resistance protein, and differences in recognition are governed by surface-exposed polymorphic residues [24], [31]. Effectors are probably also under selection to adapt to alterations in host proteins targeted by their virulence functions or to acquire new virulence targets. Comparison of effector complements from multiple rust species [17], [25] reveals some families that are widely conserved and are enriched for proteins with signatures of enzyme activity that may play general roles in virulence, e.g., as cell wall–degrading enzymes. In contrast, many candidate effectors are not conserved across genus or species boundaries [16], [17] and can be highly variable between isolates of the same species [14], [32]. This class includes known Avr proteins from flax rust and is likely to be enriched for such determinants of host specificity.

## Conclusion

The use of modern technologies to study the highly specialised dikaryotic haustorium of rust fungi has provided convincing support of the early idea that it comprises a feeding apparatus that allows the pathogen to parasitise the host. The intimate and long-lasting relationship between pathogen and plant also demands that the host immune system is dampened or disabled. Both of these functions are likely to be dependent upon the secretion of effector proteins that condition the host to accommodate the infection. Although the availability of genomes and transcriptomes of rust fungi have helped to uncover their effector coding potential, precise roles for effectors during infection is an unexplored frontier with great potential to define fascinating new aspects of biology. Thus, the development of systems to screen candidate effectors for their role in disease [33] will expand our understanding of these important proteins and increase the options to control rust pathogenic fungi.
